# An interdisciplinary guideline development process: the Clinic on Low-back pain in Interdisciplinary Practice (CLIP) low-back pain guidelines

**DOI:** 10.1186/1748-5908-2-36

**Published:** 2007-11-24

**Authors:** Michel Rossignol, Stéphane Poitras, Clermont Dionne, Michel Tousignant, Manon Truchon, Bertrand Arsenault, Pierre Allard, Manon Coté, Alain Neveu

**Affiliations:** 1 Montreal Department of Public Health, McGill University, Montreal, Canada; 2 Department of Rehabilitation, Laval University, Quebec City, Canada; 3 Department of Rehabilitation, Sherbrooke University, Sherbrooke, Canada; 4 Department of Industrial Relations, Laval University, Quebec City, Canada; 5 School of Rehabilitation, University of Montreal, Montreal, Canada; 6 Sir Mortimer B Davis Jewish General Hospital, Montreal, Canada; 7 Jewish Rehabilitation Hospital, Montreal, Canada; 8 Constance Lethbridge Rehabilitation Centre, Montreal, Canada

## Abstract

**Background:**

Evaluation of low-back pain guidelines using Appraisal of Guidelines Research and Evaluation (AGREE) criteria has shown weaknesses, particularly in stakeholder involvement and applicability of recommendations. The objectives of this project were to: 1) develop a primary care interdisciplinary clinical practice guideline aimed at preventing prolonged disability from low-back pain, using a community of practice approach, and 2) assess the participants' impressions with the process, and evaluate the relationship between participant characteristics and their participation.

**Methods:**

Ten stakeholder representatives recruited 136 clinicians to participate in this community of practice. Clinicians were drawn from the following professions: physiotherapists (46%), occupational therapists (37%), and family physicians (17%). Using previously published guidelines, systematic reviews, and meta-analyses, a first draft of the guidelines was presented to the community of practice. Four communication tools were provided for discussion and exchanges with experts: a web-based discussion forum, an anonymous comment form, meetings, and a symposium. Participants were prompted for comments on interpretation, clarity, and applicability of the recommendations. Clinical management recommendations were revised following these exchanges. At the end of the project, a questionnaire was sent to the participants to assess satisfaction towards the guidelines and the development process.

**Results:**

Twelve clinical management recommendations on management of low-back pain and persistent disability were initially developed. These were discussed through 188 comments posted on the discussion forum and 103 commentary forms submitted. All recommendations were modified following input of the participants. A clinical algorithm summarizing the guidelines was also developed. A response rate of 75% was obtained for the satisfaction questionnaire. The majority of respondents appreciated the development process and agreed with the guideline content. Most participants thought recommendations improved between versions, and that participant comments contributed to this improvement. All stakeholders officially endorsed the guidelines.

**Conclusion:**

The community of practice approach was a successful method to develop guidelines on low-back pain, with participants providing information to improve guideline recommendations. The information technology infrastructure that was developed remains for continuous interdisciplinary exchanges and updating of the guidelines.

## Background

The "Appraisal of Guidelines Research and Evaluation" (AGREE) collaboration has identified the different dimensions that a guideline should address in order to demonstrate quality and improve its effectiveness [1,2]. Several reviews have since used the tool developed by the AGREE collaboration to asses the quality of clinical practice guidelines. Reviews on knee osteoarthritis[3], low back pain[4], osteoporosis[5], lung cancer[6], and diabetes[7], essentially obtained the same results: while scope/purpose, clarity/presentation and rigour of development were adequately addressed in most guidelines, stakeholder involvement, applicability, and editorial independence were much less adequately addressed. Applicability allows guideline developers to identify and take into account barriers related to the use of the guideline, with the aim of improving usability[8]. Stakeholder involvement renders the guideline development process more transparent and facilitates appropriation by the end-users[9]. Stakeholder involvement and applicability are closely linked, since applicability is often assessed with the input from stakeholders.

Although stakeholder involvement, applicability, and editorial independence should be addressed during guideline development, the AGREE instrument and literature do not explicitly describe ways to effectively address them, apart from editorial independence, which only requires that guideline group members complete editorial independence and conflict of interest statements. In order to facilitate and structure exchanges between researchers, stakeholders, and clinicians, communities of practice (CoP) have been proposed[10]. It is a process of social learning that occurs when people with a common interest collaborate over an extended period to share ideas, solve problems, and create knowledge, such as practice guidelines [11]. It creates a meeting place for people who normally would not interact, and encourages discussion among them. Through this process, members involved in complex systems share knowledge and learn from one another, with tacit clinician knowledge considered as important as scientific knowledge [12], creating an atmosphere of cooperation and trust. It can contribute to improving both clinical practices and research [10] by focusing not only in the internal but also the external validity of the guideline [13]. A social norm of practice can result from a CoP process, reducing individual practice variations[12]. CoPs have been effectively used in various non-health settings by improving practices and productivity[12]. CoPs appear especially of interest in fragmented multidisciplinary environments by favouring long-term exchange of information and knowledge among participants[14]. Web-based technologies have been demonstrated to be efficient tools to structure CoPs among widely dispersed individuals with different work schedules[15,16].

Low-back pain (LBP) is one of the most prevalent health problems in industrialized countries, engendering significant disability and costs. Back pain will generally resolve itself in the short term, with only a minority developing prolonged disability[17]. However, this minority is responsible for the majority of costs and has the poorest health outcomes. There is also scientific consensus that predictors of prolonged disability are more psychosocial than biomedical in nature[18]. Interdisciplinarity has also been shown to be effective in addressing the multidimensional aspects of prolonged disability related to LBP[19]. Thus, a shift of clinical focus from pathophysiology to the prevention of prolonged disability is needed in primary care clinicians involved in LBP management[20].

The previous elements and the lack of guidelines in LBP management in the province of Quebec, Canada triggered a movement to bring the different stakeholders in the province to work together on the development of interdisciplinary LBP guidelines. The guidelines were to be suited to primary care clinicians (*e.g*., family physicians, physiotherapists, and occupational therapists) and contribute to better quality and continuity of care for patients with LBP. These three groups of professionals provide the vast majority of primary care treatments to workers suffering from LBP in the province.

Although CoPs are suggested as a method to improve stakeholder involvement and applicability, it is not known how this process can apply to guideline development, how participants view this process, and what participant characteristics are related to participation in the process. The objectives of this project were to develop a primary care interdisciplinary clinical practice guideline aimed at preventing prolonged disability from LBP using a CoP approach, assess the participants' impressions with the process, and evaluate the relationship between participant characteristics and their participation

## Methods

### Participants

**Figure 1:**
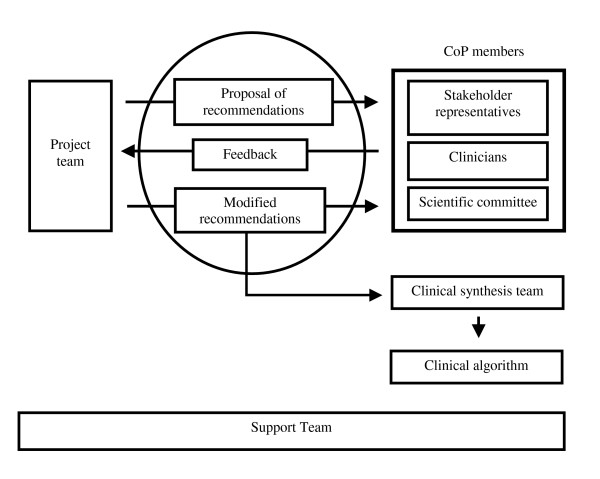
The organization of the CLIP guideline development process.

The Clinic on Low-back pain in Interdisciplinary Practice (CLIP) initiative was created and led by a project team of eight members representing research, academic, and clinical experiences: one occupational health physician researcher, two physiotherapist researchers, one occupational therapist researcher, one psychologist researcher, two family physicians, and one physiotherapist clinician. A CoP was put into place (Figure 1) to ensure interdisciplinarity in all processes that would lead to the endorsement of clinical guidelines on LBP by all stakeholders. Key stakeholders included representatives from the family physician, physiotherapy, occupational therapy licensing boards, and clinician associations of the province, along with observers from the Quebec Workers' Compensation Board and its research institute, the Institut de Recherche Robert-Sauvé en Santé et en Sécurité du Travail (IRSST). Stakeholder representatives were asked to identify and invite members throughout the province who were recognized as experts, opinion leaders, or who had an interest in LBP management to participate in this CoP. The project team also formed a seven-member scientific committee composed of researchers from different universities and disciplines (orthopedics, occupational therapy, physiotherapy, epidemiology, rheumatology, and anthropology), with the objective of independently evaluating the content of the guidelines. Finally, a clinical synthesis team was formed by the project team, who identified and invited three physicians, three physiotherapists, and three occupational therapists recognized as opinion leaders in LBP management. Their task was to summarize the guidelines recommendations in the form of a clinical algorithm. The CoP was supported by experts in literature evaluation and synthesis, group animation, communications, scientific editing and illustration, web-based technologies, and administration.

### Guideline development process

The UK Royal College of General Practitioners (RCGP) LBP guideline[21] published in 2001 was used as the starting point. It was selected because it is a primary care multidisciplinary guideline of relatively high quality[4]. The literature review was updated to 2005 using the Medline, Embase, and Cochrane libraries for systematic reviews, meta-analyses, and key randomized controlled trials. The goal of the review was to identify potential shifts in findings since the 2001 RCGP guideline. The general layout of the guidelines was divided in three sections: evaluation of the LBP patient, therapeutic approach of LBP, and management of prolonged pain and disability. Each section contained specific recommendations. Each recommendation was written by the project team members on a maximum of one page, including a recommendation statement, a grading of strength of evidence, a brief description of scientific evidence in support of the recommendation, an interpretation in terms of best practice options, and a short list of references selected for educational purposes. Examples of tools to apply the recommendations, such as questionnaires, were also provided.

Each recommendation was presented to the CoP by postal mail, e-mail, and website simultaneously. The presentation of each section was done sequentially, in order to allow at least one month of discussion and exchanges among participants. Section one was released in September 2004, section two in March 2005, and section three in September 2005. Two web-based communication mechanisms were offered to the participants to discuss the recommendations: an open online discussion forum, and an online commentary form with closed and open questions that could be sent confidentially. Members of the project team were asked to moderate the discussion forums. Additionally, the recommendations were discussed at a mid-point symposium (April 2005).

Comments received on the website, at the mid-point symposium and from the scientific committee, were systematically analyzed for their content. Taking into account these comments, project team members decided by consensus if and how the recommendations should be modified without deviating from the evidence. If there was no consensus on a specific issue, the divergence of interpretation was reflected in the revised text. The revised recommendations were then released in October 2005. The revised recommendations were provided to the clinical synthesis team with the mandate of preparing a clinical algorithm for the guideline. For this, they met until there was a consensus on the content and format of the algorithm. This task was accomplished over two days in February 2006. The final version of the guideline was released at the last symposium in April 2006.

### Satisfaction of participants with the CLIP guidelines and process

Three months after the release of the final version of the guideline, an online survey was sent to the stakeholder representatives and the extended group of clinicians in order to obtain their evaluation of the CLIP guidelines and process. The questionnaire contained 18 items on guideline content and the development process, assessed on a five-point Likert scale and respondents' characteristics. The questionnaire was sent by e-mail, with reminder letters sent by postal mail. The relationships between the survey results and respondent characteristics (age, years of practice, and profession) were assessed with chi-square and Kruskal-Wallis tests. A level of significance of 0.05 was used for all analyses.

## Results

Participating clinicians included 136 individuals, 62 (46%) of which were physiotherapists, 51 (37%) occupational therapists, and 23 (17%) family physicians. There were ten stakeholder representatives. Twelve recommendations for the management of LBP were initially developed by the project team. From the release of section one to the last comment posted on the revised recommendations (total of 15 months), the website was visited 3,758 times, with 188 comments posted on the discussion forum and 103 commentary forms submitted. Forty-seven participants came to the mid-term symposium and 95 were present at the final symposium, which was open not only to CLIP participants but to all interested clinicians.

Comments from the participants were made on the following subjects: clarity (objectives pursued, use of evaluations, and interventions), agreement, coherence among recommendations, completeness, compatibility with current practice and knowledge, competencies needed, applicability with clientele, impact on patient's health and satisfaction, usability (taking into account resources, health care organization and laws), perceptions and practices of colleagues and other professionals, and elements and tools needed for successful implementation. Members of the scientific committee additionally provided comments on the validity of the recommendations.

During this process, all recommendations were either reorganized or modified, ranging from minor rewording to extensive conceptual modifications. For example, recommendation 1.2 was modified from "Radiographic, MRI or CT scan examinations are not indicated for patients with simple back pain" to "Radiographic, MRI or CT scan examinations are rarely indicated...". This was a topic that triggered much debate in the confrontation of scientific evidence and clinical practice. Finally, an algorithm summarizing and linking the final recommendations through the different stages of LBP was developed by the clinical synthesis team.

### Satisfaction with the CLIP guidelines and development process

The questionnaire to assess satisfaction towards the CLIP process and the guidelines was sent to the 146 participants. The questionnaire was completed by 110 participants, seven declined to participate (5%) and 29 did not reply (20%), for a response rate of 75%. Response rate was significantly lower for physicians. Table 1 describes the respondents' characteristics, while tables 2 and 3 summarize their answers. The majority of the respondents reported having actually participated in the CLIP process (n = 78;53%) or read the final guidelines (n = 69;47%). Lack of time was by far the most frequent reason for non-participation (70.5% of reasons). Among those who reported having participated, level of participation was variable.

**Table 1:** Characteristics of the respondents to the CLIP questionnaire (n = 110)

	**N (%)**	**Missing N (%)**
	
Female gender	68 (62%)	6 (5%)
Mean age	38.6 years (SD: 8.9)	10 (9%)
Practicing clinician	89 (81%)	6 (5%)
Mean years of practice	14.4 years (SD: 8.7)	2 (2%)
Working in private practice	63 (71%)	2 (2%)

Profession		
Physiotherapist	50 (45%)	6 (5%)
Occupational Therapist	41 (37%)	
Family physician	12 (11%)	
Other	1 (1%)	

**Table 2:** Survey results of the respondents on the CLIP process (n = 78)

**CLIP process comments**	**N (%)**				
	**Little (1–2)**	**3**	**Very (4–5)**	N/O*	**Missing**
	
Intensity of participation	43 (55%)	20 (26%)	11 (14%)	-	4 (5%)
Appreciation of CLIP elements:					
Discussion forums	14 (18%)	15 (19%)	33 (42%)	12 (15%)	4 (5%)
Anonymous questionnaires	8 (10%)	16 (21%)	17 (22%)	32 (41%)	5 (6%)
CLIP symposia	5 (6%)	4 (5%)	39 (50%)	26 (33%)	4 (5%)
Overall CLIP process	6 (8%)	13 (17%)	52 (67%)	3 (4%)	4 (5%)
Improvement between initial and final versions of recommendations	7 (9%)	20 (26%)	33 (42%)	13 (17%)	5 (6%)
Influence of participant comments on final recommendations	9 (12%)	18 (23%)	25 (32%)	22 (28%)	4 (5%)
Opportunity to intervene in the CLIP process	13 (17%)	12 (15%)	40 (51%)	8 (10%)	5 (6%)
	**Never**	**1–5**	**6–10**	**>11**	**Missing**
	
Frequency of CLIP website visits	5 (6%)	32 (41%)	15 (19%)	22 (28%)	4 (5%)

* No opinion

**Table 3:** Survey results of the respondents on the CLIP guideline (n = 69)

**CLIP guideline comments**	**N (%)**			
	**Little (1–2)**	**3**	**Very (4–5)**	N/O*
	
Agreement with final version of recommendations	4 (6%)	9 (13%)	55 (80%)	1 (1%)
Acquisition of new knowledge	19 (28%)	29 (42%)	20 (29%)	1 (1%)
Modification of perceptions in LBP management	28 (41%)	28 (41%)	11 (16%)	2 (3%)
Dissemination of the guideline in entourage	15 (22%)	16 (23%)	37 (54%)	1 (1%)
Importance of instating a process of continual improvement of the guideline	4 (6%)	5 (7%)	59 (86%)	1 (1%)

* No opinion

Overall, the CLIP process appeared to have been appreciated by the majority of respondents. Among the communication tools provided, the discussion forums on the website appeared to have been the most often used, while the symposia and anonymous questionnaires appeared to have been less used, according to the proportion of respondents having an opinion on them. Conversely, the symposia appeared to have been more appreciated than the web-based tools (discussion forums and anonymous questionnaires). Most respondents found that there was an improvement between the initial and final versions of the recommendations, and that the participants' comments contributed to this improvement. The majority thought they had sufficient opportunities to provide comments during the CLIP process.

As for the guidelines, the majority agreed with their content. New knowledge acquisition appeared variable among respondents, while modification of perceptions regarding LBP management appeared relatively low. Finally, the majority reported having distributed the guideline to their colleagues, and demonstrated interest in the creation and participation in a process aimed at continually updating the guidelines.

### Relationship between survey results and respondent characteristics

Only the following relationships were significant between survey results and respondent characteristics: the final version of the guidelines was less read by family physicians, while occupational therapists read it more. Older respondents reported having participated more intensely in the CLIP process. Paradoxically, they felt the participants' comments had less influenced the final version of the guidelines. Finally, occupational therapists tended to agree significantly more with the guideline content. All other relationships were not significant.

## Discussion

Studies have shown variable adherence of primary care clinicians to scientific evidence in the clinical management of LBP [22-24], which fosters the need for guidelines. The CLIP guidelines were designed for all three groups of primary care health professionals, irrespective of their specific expertise. Their goal was to promote the use of similar tools and a common language in the management of LBP from a bio-psycho-social perspective.

This guideline development process showed that CoP principles can be successfully applied in this context. One of the main focuses of this project was to encourage participation and contribution of end-users and stakeholders in the guideline development process, in order to improve validity, applicability, acceptability, appropriation, and ultimately use of the guidelines. This was archived with the participation of a relatively large sample of clinicians and stakeholders, the majority positively evaluating the development process and content of the guidelines. A substantial amount of information was exchanged among participants during the 15 months of the CoP. The community was dynamic throughout the process, and very few motivating strategies were initiated by the project team. The various types[25] and frequency [26] of communication strategies used may have encouraged these exchanges. According to the survey results regarding the element where discussion occurred (forums and symposia), sharing of ideas and opinions appeared to have been appreciated by the majority of participants. Most participants thought that the process improved the guidelines, and that participant comments contributed to this improvement. A majority of participants also reported disseminating the guidelines, a possible consequence of their appreciation of the process and guidelines. A social norm was also initiated, as all stakeholders officially endorsed the guidelines and posted them on their respective websites.

The discussion tools provided in this project did not appear to be used and valued to the same extent. Web-based discussion forums were the most often used, but symposia were the most valued. This probably highlights the strengths and weaknesses of each method, and the importance of combining several communication methods when collaborating with clinicians and stakeholders. Subjects throughout the province were easily reached through web-based discussion forums, but they did not provide the rich and diversified information accorded by face-to-face meetings in symposia. It was striking to notice the difference of dynamics between the symposia and the web-based discussion forum, the former leading to more diversified ideas, because the discussions on the website tended to be monopolized by a minority of individuals. Moderating became a challenge even with coaching by experts in the field. Web-based technologies have significant potential for guideline development, but further research is needed in order to effectively use these tools.

Other CoP principles were less successful. Input was not evenly distributed among participants. If it is presumed that non-respondents to the survey did not participate in the process, only half of the recruited participants actually participated. Participation was also skewed, with only a minority participating heavily in the process. Survey results seemed to show that older participants contributed more frequently to the CLIP discussions, possibly making less room for younger participants. However, it is not clear if older participants felt their opinions were heard, because they were less enthusiastic regarding the impact of participants' comments on the guidelines. As for participation of general practitioners, it was especially low despite considerable effort by the project team. Lack of time was the reason most often given for not participating. Facilitating physician participation in research remains a challenge [27].

Although guideline implementation was not the study objective, it appears the process had a limited impact on behavioral changes of clinicians. According to survey results, agreement with recommendations and acquisition of new knowledge by participants was higher than modification of perceptions. The difficulty of integrating knowledge related to LBP management has been previously demonstrated[28]. Perhaps integration of the guidelines would be easier for occupational therapists, because they tended to agree more with them. This result is not surprising because occupational therapists are traditionally trained following a bio-psycho-social model, as is proposed in the CLIP guideline. Adherence to guideline recommendations is influenced by numerous clinician, patient, and environmental factors, including organizational structures, policies, and laws[29,30]. This CoP process only addressed some of these, such as end-user involvement, transmission of knowledge, validity, clarity, applicability, agreement, participation of opinion leaders, transparency, legitimacy, and social norm. It is therefore expected that further strategies targeting other barriers will be needed to achieve behavior change.

The extensive stakeholder involvement had an unexpected consequence regarding future updating of the guidelines. Several stakeholders and participants mentioned at the end of the project that they expected to be contacted and involved when the guidelines would be updated. This is probably a positive outcome of the shared creation process and a sign of appropriation of the guidelines by participants and stakeholders[9]. The CoP process would have to be re-established in order to update the guidelines, challenging its long-term cost-effectiveness. Successful stakeholder involvement brings up the question of who owns the guidelines, and who is actually responsible for their update.

It could be argued that stakeholder involvement was restrictive, because participants were not involved from the start during the initial elaboration of the guideline recommendations. However, the interdisciplinary project team responsible for this initial elaboration was assembled in order to represent different clinical, academic, and research views. Also, the CoP did not limit the number of participants and was opened to the diversity of clinical and research experiences. This openness favored trust, transparency, and legitimacy of the end-product [10].

This process not only provided data to improve guideline recommendations, but also on the barriers and facilitators related to their application – data that can be used in the elaboration of future implementation strategies. Future research evaluating the conditions of implementation of the CLIP guidelines in various clinical, organizational, and geographical settings should be carried out.

## Conclusion

This study proposed a guideline development process focusing on stakeholder involvement and applicability, a process that can be transferred to other fields. The CoP approach was a successful method to develop guidelines on low-back pain, with participants providing information to improve the validity and applicability of guideline recommendations. The majority of participants appreciated the development process and agreed with the guideline content. All stakeholders officially endorsed the guidelines. The CLIP guidelines are available on the internet[31].

## Competing interests

The author(s) declare that they have no competing interests.

## Authors' contributions

All authors participated in conception and design of the study, acquisition of data, interpretation of data, and revision of the manuscript. All authors read and approved the final manuscript. MR and SP additionally analyzed the data and drafted the manuscript
